# Short-Term Statistical Forecasts of COVID-19 Infections in India

**DOI:** 10.1109/ACCESS.2020.3029614

**Published:** 2020-10-08

**Authors:** Ram Kumar Singh, Martin Drews, Manuel De La Sen, Manoj Kumar, Sati Shankar Singh, Ajai Kumar Pandey, Prashant Kumar Srivastava, Manmohan Dobriyal, Meenu Rani, Preeti Kumari, Pavan Kumar

**Affiliations:** 1 Department of Natural ResourcesTERI School of Advanced Studies New Delhi 110070 India; 2 Department of TechnologyUniversity of Denmark 2800 Kongens Lyngby Denmark; 3 Institute of Research and Development of Processes IIDP, University of the Basque Country Campus of Leioa 48940 Spain; 4 GIS CentreForest Research Institute (FRI), PO: New Forest Dehradun 248006 India; 5 Extension EducationRani Lakshmi Bai Central Agricultural University Jhansi 284003 India; 6 College of Forestry and HorticultureRani Lakshmi Bai Central Agricultural University Jhansi 284003 India; 7 Institute for Environment and Sustainable DevelopmentBanaras Hindu University 30114 Varanasi 221005 India; 8 Department of GeographyKumaun University 30102 Nainital 263001 India; 9 Department Environmental Science and EngineeringIndian Institute of Technology Dhanbad 826004 India

**Keywords:** COVID-19, forecasts, GIS, health services, holt-winters, India, SIR model

## Abstract

COVID-19 cases in India have been steadily increasing since January 30, 2020 and have led to a government-imposed lockdown across the country to curtail community transmission with significant impacts on societal systems. Forecasts using mathematical-epidemiological models have played and continue to play an important role in assessing the probability of COVID-19 infection under specific conditions and are urgently needed to prepare health systems for coping with this pandemic. In many instances, however, access to dedicated and updated information, in particular at regional administrative levels, is surprisingly scarce considering its evident importance and provides a hindrance for the implementation of sustainable coping strategies. Here we demonstrate the performance of an easily transferable statistical model based on the classic Holt-Winters method as means of providing COVID-19 forecasts for India at different administrative levels. Based on daily time series of accumulated infections, active infections and deaths, we use our statistical model to provide 48-days forecasts (28 September to 15 November 2020) of these quantities in India, assuming little or no change in national coping strategies. Using these results alongside a complementary SIR model, we find that one-third of the Indian population could eventually be infected by COVID-19, and that a complete recovery from COVID-19 will happen only after an estimated 450 days from January 2020. Further, our SIR model suggests that the pandemic is likely to peak in India during the first week of November 2020.

## I. Introduction

The SARS-CoV-2, i.e. severe acute respiratory syndrome coronavirus disease 2 (COVID-19) has to date (27 September 2020) infected more than 33 Million people worldwide, caused almost one million mortalities, and forced more than 10000 Million people to stay within their homes [1], [2]. Due to the dynamics of COVID-19 transmission, which is still to be fully understood by researchers, and the high number of infected people globally, managing the associated health risks is still the main priority and historically affects the economic activities of many countries [3]–[8]. The first cases of COVID-19 were reported in December 2019 in Wuhan in China [9]–[11]. Already by February 2020, it had spread to large parts of the world, and as of 12 September 2020, the number of people infected rounded 33.23 million. Due to this rapid pandemic potential and the current absence of antiviral drugs and vaccines, COVID-19 has placed a tremendous strain on essential health services and left medical personnel to cope with case numbers critically exceeding capacities. For example, in India, where COVID-19 cases have been steadily increasing since January 30, 2020. Despite a government-imposed lockdown across the country to curtail community transmission, the number of cases in India continues to soar [12]–[15].

Mathematical-epidemiological models are widely used to infer critical epidemiological transitions and transmission parameters of COVID-19. Such models aim to predict the spread of infectious diseases, their implications and potentially also the effect of preventive measures [16]. Methods used include epidemic curve fitting, classical epidemiological compartment models like the SIR (Susceptible, Infectious, and Recovered) model and derivatives thereof, and statistical time series models [17]–[19]. In forecasting mode, such models often rely on monitoring data obtained during earlier transmission stages and based thereof can be used to predict the development of COVID-19 pandemic across the world on different time scales.

As demonstrated, e.g., in Europe, skillful model forecasts [20], [21] are urgently needed to identify the most effective and sustainable strategies for coping with COVID-19 at the local or regional level. Due to lack of data and availability of local epidemiological models, in many instances, however, access to up to date forecast information is surprisingly scarce considering its evident importance. This includes India, where authorities and academia generally collect data and provide forecasts based on state-of-the-art epidemiological models at the national level [22]. Such nationwide forecasts are not easily transferable/ scalable to the level needed by the regional authorities and/ or health services in India, who has to make critical decisions on mitigating measures and resource allocation in the light of unprecedented COVID-19 pressures [23], [25]. Here, local, short-term forecasts for example contribute to the strategic planning for coping with the increased hospital needs due to COVID-19 [26], [27].

The Holt-Winters family of statistical methods derives from classical non-linear time series analysis. It is essentially based on a triple exponential smoothing, accounting for level, trend and seasonality in a time series [28]. The Holt-Winters model is integrated into most standard statistical software, and thereby available for most users unlike more complicated and realistic models requiring special expertise.

In the following, we demonstrate the performance and limitations of the Holt-Winters method as means of providing short-term COVID-19 forecasts in India based on observed records of infections. This approach is fully data-driven [29] and thereby easily adaptable for regional and local usage. We demonstrate its capabilities using nation-wide data series. These can easily be replaced by similar regional data series for dedicated use at the state, regional or even city level. Lastly, we compare with and discuss the longer term perspective of COVID-19 infections in India, based on a simple implementation of the “iconic” SIR model.

## II. Material and Methodology

### A. Holt-Winters Method Data Replications

**FIGURE 1.**
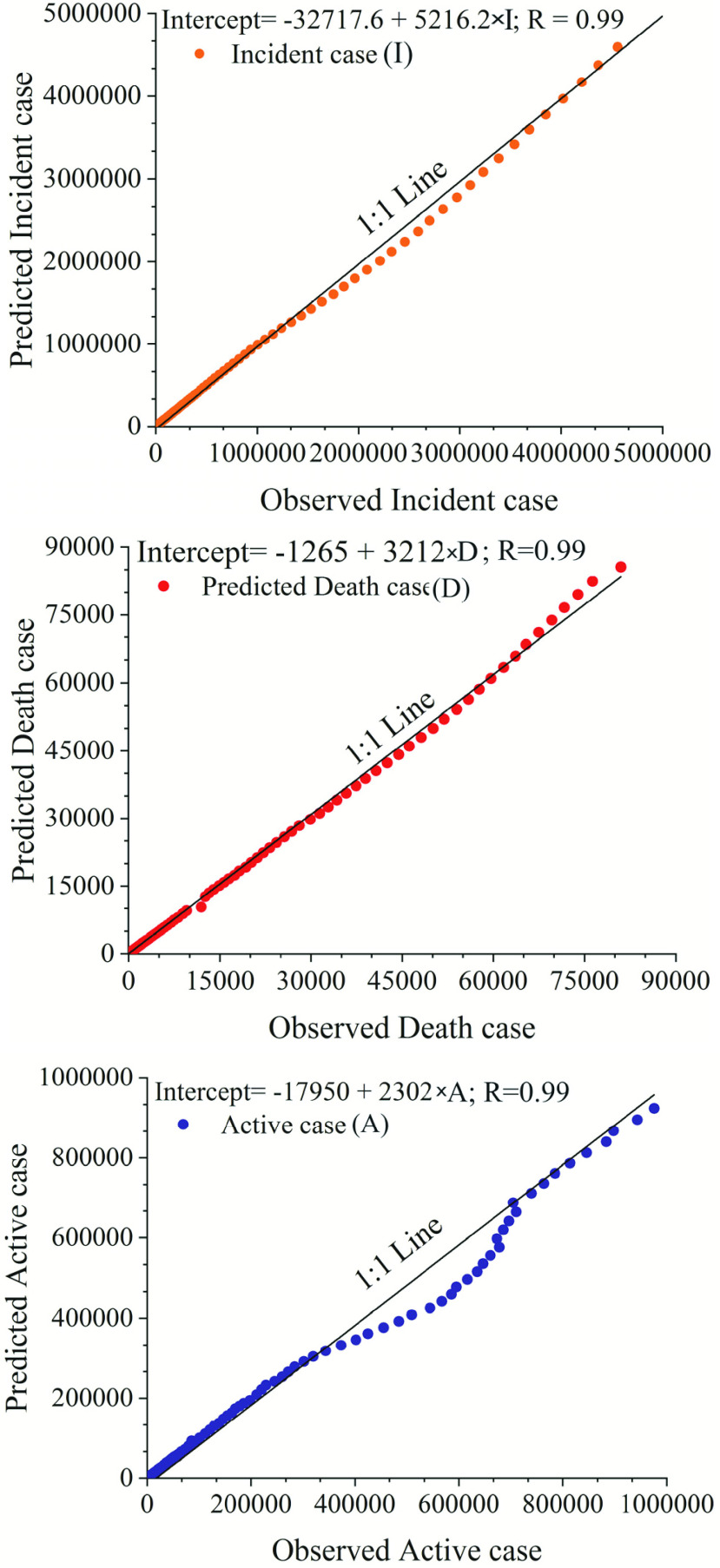
Observed vs. predicted COVID-19 cases in India obtained using a Holt- Winters statistical model for the period of $3^{\mathbf {rd}}$ July, 2020 to $12^{\mathbf {th}}$ September, 2020. The regression lines correspond to the Pearson correlation (R). The panels (from top to bottom) correspond to the (cumulative) confirmed infections, (cumulative) deaths and active cases, respectively.

For our statistical modelling, we extracted daily time series data for India collected by the Johns Hopkins Corona Virus Resource Center [30], Worldometer Covid-19 [31] and Mygov-Government of India [32] from 22 January 2020 to 27 September 2020. Our data-driven forecast model for the spread of COVID-19 in India employs the non-linear Holt-Winters method [33] as implemented in the R statistical software (R Core Team 2013). We trained our forecast model on the initial 250 days of our time series. For validation assessments the following 70 days, i.e., the period from 03 July 2020 to 12 September 2020 was used. We calculated the Mean Absolute Percentage Error (MAPE) for three predicted compartments, i.e., cumulative confirmed infections, active cases, and cumulative deaths (see Table 1) in India. Figure 1 shows a detailed comparison between the predicted and observed daily values for the validation period. In all three cases, the skill of the Holt-Winters models proved to be reasonably good. Further model validation was carried out by calculating Pearson’s correlation coefficient and the associated p-value, i.e., comparing predicted and observed cases. We here used the Kwiatkowski– Phillips–Schmidt–Shin (KPSS) statistical test to test the null hypothesis. In all cases, the p-value was found to be less than 0.01, indicating a very good agreement between the statistical model and observations.

**TABLE 1:** Predictive Performance of the Statistical Forecasts Measured by Mean Absolute Percentage Error (MAPE)

Prediction	Confirmed cases	Deaths	Active cases
MAPE	11.2298 %	16.5113 %	13.2542 %

**FIGURE 2.**
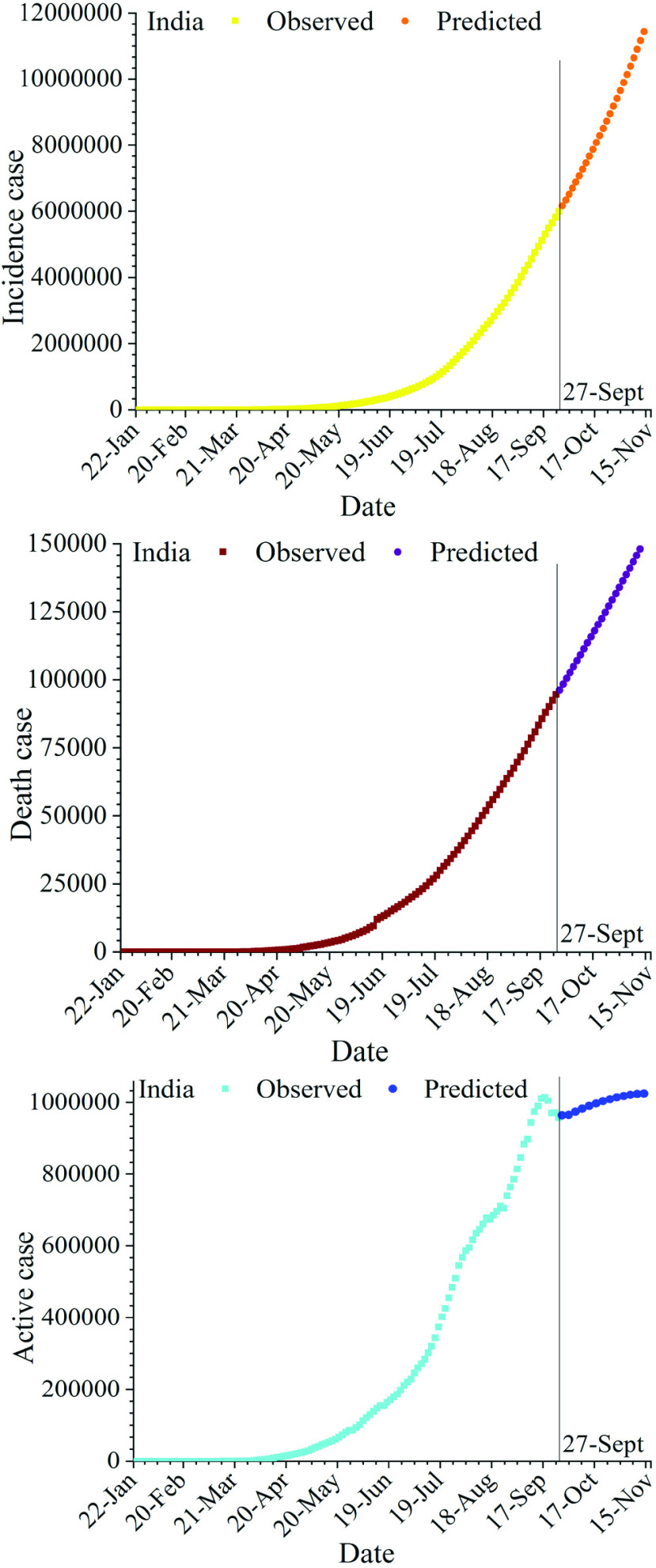
Predictions 48 days ahead (28 September to 15 November 2020) of (cumulative) (a) infections, (b) deaths and (c) recoveries, respectively; from COVID-19 in India using a Holt-Winters forecast model (shading indicates the 95% confidence interval).

For demonstration purposes, Figure 2 shows analogous 48-day forecasts (28 September to 15 November) using the Holt-Winters method and trained on the full time series up to and including 27 September, 2020.

### B. Susceptible-Infectious-Recovered (SIR) Model

**FIGURE 3.**
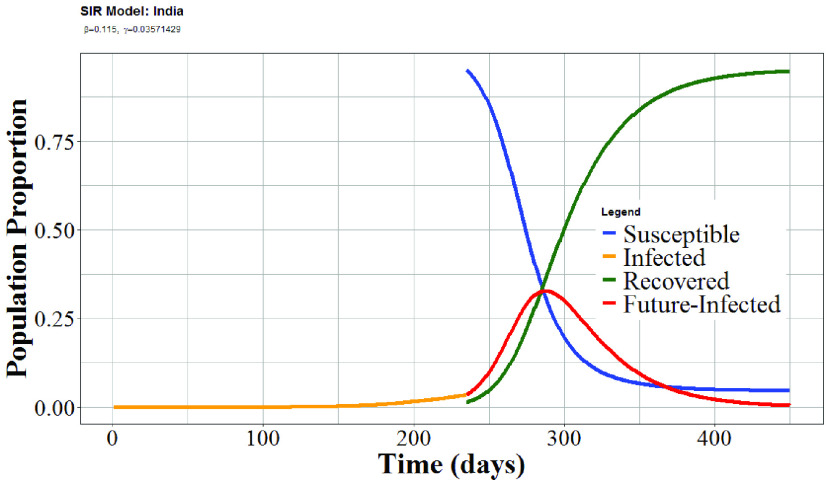
SIR model of COVID-19 pandemic in India, representing the Susceptible, Infected, and Recovered cases as a function of the number of days since 22 January 2020. The model shows a peak around 290 days (06 November 2020) and complete recovery after four hundred and fifty days from 22 January 2020.

For comparison, we also implemented a classical mathematical-epidemiological compartment model, the Susceptible-Infectious-Recovered (SIR) model, which has already been explored by several authors for COVID-19 [34]. The basic form of the SIR model consists of three linked differential equations and considers transitions between three population compartments as a function of time: infected, susceptible and recovered. The transitions are governed by two parameters: the transmission rate ($\beta$) and the recovery rate ($\gamma$). The model transmission rate ($\beta$) is the proportion of the population that is infected and show symptoms within five days. Here we use $\beta = 0.1152$. The recovery rate ($\gamma$) proposed by WHO [35] is between two to six weeks depending on the necessary medical support. In the model used here, we assume it is four weeks ($\gamma = 1$/28). In our model simulations, we tested three different assumptions with regards to the number of people in the susceptible compartment. Firstly, we assumed that the population of India ($N$) is closed and homogenous (i.e. we disregard new births and deaths) and let that define the susceptible compartment. Second, we assumed that the homogenous population only fluctuated by means of COVID-19 influence (deaths). In the third case, susceptible individuals are infected, and the compartments are updated dynamically. These assumptions proved to have very little influence on the COVID-19 projections and hence in the following we highlight only the third scenario. According to these different assumptions regarding the size of the susceptible population, transmission and recovery rates, we use the basic form of the SIR model to estimate the temporal development of COVID-19 in India for the three compartments (Figure 3) since 22 January, 2020.

## III. Result and Discussion

Figure 2 shows the predicted trajectories corresponding to each of the three cases (cumulative confirmed infections, cumulative deaths and active cases) up to 15 November 2020. In this period, the number of cumulative confirmed COVID-19 cases in India in the period is expected to rise rapidly to an estimated 12,132,049 cases. Similarly, cumulative deaths are expected to toll just above 150,639, whereas the numbers of active cases are predicted to increase to more than 1025035 cases. The rapidly increasing number of active cases predicted statistically is likely to have significant impacts on the Indian health system, although obviously with large regional and local variations. Meanwhile COVID-19 cases resulting in deaths are depicted to show less dramatic increases, and even a slight flattening of the overall curve is observed. Like the two other curves, the recovery rate is seen to increase (significantly) over the period.

We can further extrapolate from these predictions by using them in a SIR model as mentioned above [36] (see Figure 3). Comparing the two model forecasts for the 48-day period shown in Figure 2, the basic SIR and Holt-Winters model predictions roughly agrees. As mentioned above, our statistical predictions indicate that the numbers of COVID-19 cases in India are going to rise at alarming rates in the short term.

On the longer term, from the SIR model analysis, one would infer weak signs that that the spread of COVID-19 in India is nearly peaking. From the SIR model, we find that without further preventive measures the pandemic will peak in India around 290 days (06 November) from 22 January, where about one-third of the Indian population will have been infected at some point. After this, it will gradually decrease. Similarly, we see predicted declines in the number of cases leading to mortalities and an increasing trend over time with respect to recoveries. The SIR model indicates a complete recovery will be achieved after 450 days from the January 22 2020, if not any medical and administration level of interventions will be achieved. This result should be considered for academic discussions only. Hence, the estimated model parameters used in this paper are easily up for discussion and improvements. Moreover, there is no comprehensive scientific evidence for the skill of the basic SIR model for long-term COVID-19 forecasts. The same applies to advanced versions of the SIR model. Hence, the Indian health services and capacity should continue to be on their toes, as this will be critical important in order to gain control of COVID-19 in India.

**Limitations of the study**

As mentioned above, the Holt-Winters method stems from time series analysis. The method aims to capture the level, trend and seasonality of a time series through exponential smoothing, and based thereupon to make predictions. Like all purely statistical models, it learns only from observations and do not include epidemiological knowledge, and hence predictions are made solely based on recently observed trends and seasonality, although the latter does not apply to COVID-19. For this reason, this kind of model is unable to represent or account for abrupt trend changes caused by, e.g., successful actions towards limiting the spread of COVID-19. Accordingly, we here only use this technique to model short- term (48-day) developments, where the base assumption of “stationarity” is likely to hold and discourage its use for modelling longer-term developments.

In this study, we use the basic form of the SIR model, which is arguably too simplistic to provide realistic COVID-19 predictions, in particular for the longer-term. For this aim, several authors have proposed more advanced compartment models, derived from the SIR model. That said, despite a world-wide scientific focus on COVID-19, there is still a lot we don’t know about the virus [37] and therefore it is also impossible to quantify the skill of any model. As mentioned above, we here use the SIR model results as means of qualifying the short-term forecasts provided by the Holt-Winters method, for stimulating academic discussion, and we strongly discourage their use for real-life planning purposes.

## IV. Conclusion

This paper demonstrates the performance of short-term statistical forecasts using Holt-Winters method and suggests that this method could be suitable for providing operational COVID-19 forecasts in India aimed at different administrative levels. Hence, the Holt-Winters method is integrated into most statistical software, making it readily available to non-experts from outside the mathematical-epidemiological modelling community. 48-day re-forecasts of cumulative infections, cumulative deaths and active cases in India based on a trained Holt-Winters model reproduce the observed values reasonably well. For a future period, Holt-Winters forecasts are found to be comparable to those of a basic SIR model.

In general, in cases such as this one, where there is no seasonal signal involved, Holt-Winters models capture the level and most recent trend of a time series and as such are unsuited for long-term forecasts. For such forecasts, more advanced mathematical-epidemiological models are needed, and in the paper we illustratively show the results of a SIR model, indicating that the number of COVID-19 infections will peak by November 2020.
